# Differential effects of FTY720 on the B cell compartment in a mouse model of multiple sclerosis

**DOI:** 10.1186/s12974-017-0924-4

**Published:** 2017-07-24

**Authors:** Kathrin Bail, Quirin Notz, Damiano M. Rovituso, Andrea Schampel, Marie Wunsch, Tobias Koeniger, Verena Schropp, Richa Bharti, Claus-Juergen Scholz, Konrad U. Foerstner, Christoph Kleinschnitz, Stefanie Kuerten

**Affiliations:** 1 Department of Anatomy and Cell Biology, University of Würzburg, Würzburg, Germany; 2 Core Unit Systems Medicine, University Hospitals of Würzburg, Würzburg, Germany; 3 LIMES Institute, University of Bonn, Bonn, Germany; 4 Department of Neurology, University Hospital Würzburg, Würzburg, Germany; 5 Department of Neurology, University Hospital Essen, Essen, Germany; 6 Institute of Anatomy and Cell Biology, Friedrich-Alexander University Erlangen-Nürnberg, Erlangen, Germany

**Keywords:** B cells, EAE, Fingolimod, FTY720, Multiple sclerosis, TLO

## Abstract

**Background:**

MP4-induced experimental autoimmune encephalomyelitis (EAE) is a mouse model of multiple sclerosis (MS), which enables targeted research on B cells, currently much discussed protagonists in MS pathogenesis. Here, we used this model to study the impact of the S1P_1_ receptor modulator FTY720 (fingolimod) on the autoreactive B cell and antibody response both in the periphery and the central nervous system (CNS).

**Methods:**

MP4-immunized mice were treated orally with FTY720 for 30 days at the peak of disease or 50 days after EAE onset. The subsequent disease course was monitored and the MP4-specific B cell/antibody response was measured by ELISPOT and ELISA. RNA sequencing was performed to determine any effects on B cell-relevant gene expression. S1P_1_ receptor expression by peripheral T and B cells, B cell subset distribution in the spleen and B cell infiltration into the CNS were studied by flow cytometry. The formation of B cell aggregates and of tertiary lymphoid organs (TLOs) was evaluated by histology and immunohistochemistry. Potential direct effects of FTY720 on B cell aggregation were studied in vitro.

**Results:**

FTY720 significantly attenuated clinical EAE when treatment was initiated at the peak of EAE. While there was a significant reduction in the number of T cells in the blood after FTY720 treatment, B cells were only slightly diminished. Yet, there was evidence for the modulation of B cell receptor-mediated signaling upon FTY720 treatment. In addition, we detected a significant increase in the percentage of B220^+^ B cells in the spleen both in acute and chronic EAE. Whereas acute treatment completely abrogated B cell aggregate formation in the CNS, the numbers of infiltrating B cells and plasma cells were comparable between vehicle- and FTY720-treated mice. In addition, there was no effect on already developed aggregates in chronic EAE. In vitro B cell aggregation assays suggested the absence of a direct effect of FTY720 on B cell aggregation. However, FTY720 impacted the evolution of B cell aggregates into TLOs.

**Conclusions:**

The data suggest differential effects of FTY720 on the B cell compartment in MP4-induced EAE.

**Electronic supplementary material:**

The online version of this article (doi:10.1186/s12974-017-0924-4) contains supplementary material, which is available to authorized users.

## Background

Multiple sclerosis (MS) is a neuroinflammatory and demyelinating disease of the central nervous system (CNS) that leads to irreversible neurological deficits and premature retirement predominantly in young adults [1]. Still today, the etiology of the disease has remained unclear, but an interaction between genetic, environmental, and infectious agents is assumed to play a role in disease initiation and maintenance [1]. For decades, autoreactive T cells were primarily thought to mediate MS [2]. However, an additional crucial role of B cells in the pathogenesis of MS is underlined by clinical as well as immunological observations. B cell depletion with anti-CD20 antibodies ameliorated both clinical symptoms and magnetic resonance imaging (MRI) activity in MS patients [3–5]. Therapeutic plasma exchange was more likely to be beneficial in patients with MS lesions that showed complement and immunoglobulin depositions [6]. CNS-reactive B cells were detected in the peripheral blood of MS patients [7] and clonally expanded B cells were found in the CNS and cerebrospinal fluid (CSF) [8]. Along these lines, oligoclonal bands (OCBs) indicate intrathecal antibody synthesis [2] and antibody depositions with concurrent complement activation represent the most frequently observed pattern of demyelination in MS brain lesions [9]. We have introduced a B cell-/antibody-dependent model of experimental autoimmune encephalomyelitis (EAE), which is based upon active immunization of C57BL/6 (B6) mice with MP4, a fusion protein of myelin basic protein (MBP) and proteolipid protein (PLP) [10]. One of the hallmark features of the model is the development of B cell aggregates mainly in the cerebellum, which evolve into tertiary lymphoid organs (TLOs) in the chronic stage of the disease and resemble secondary lymphoid organs (SLOs) both in morphology and function [11]. Similar findings were recently reported in a spontaneous model of EAE showing that B cells actively participate in the CNS immune response in meningeal ectopic lymphoid tissue [12]. B cell aggregates have also been described in the meninges of post mortem brain sections of patients with secondary progressive MS (SP-MS) [13] and were associated with an early disease onset, a severe disease course and cortical histopathology [14, 15]. Unfortunately, treatment options for SP-MS are very limited and therefore approved drugs for relapsing-remitting MS (RR-MS) are currently re-evaluated in ongoing studies [16]. FTY720, also known as fingolimod, was the first oral drug for the treatment of RR-MS. FTY720 significantly reduces relapse rates and gadolinium-enhancing MRI lesions [17]. Pharmacologically, FTY720 is a S1P_1_ receptor modulator and traps lymphocytes in SLOs, resulting in a reduction of inflammatory cells both in the circulation and CNS [18]. In addition, other S1P_1_ subtype receptor-mediated effects on oligodendrocytes, astrocytes, neurons, microglia, or on the blood-brain-barrier (BBB) via interaction with endothelial cells were discussed [19]. These potential effects of FTY720 included reduced leakiness of the BBB, stimulation of astrocyte migration or the promotion of oligodendrocyte precursor cell survival [19]. Furthermore, sphingolipids seem to play an important role in neurogenesis and remyelination, and therefore, FTY720 may also influence these pathways [19]. However, so far, there is no evidence for an impact of FTY720 on B and T lymphocyte effector function [20]. The aim of this study was to evaluate the effect of FTY720 on autoreactive B cells in the MP4-EAE model in terms of autoantibody production, B cell subset distribution and the formation of B cell aggregates in the CNS. Our results demonstrate that only an early application of FTY720 was capable of ameliorating clinical disease and preventing the formation of B cell aggregates in vivo. Drug administration during the chronic stage of the disease remained ineffective on these parameters, but displayed an inhibitory effect on B cell aggregate transformation into TLOs.

## Methods

### Mice

Female 6-week-old B6 mice were purchased from Envigo (The Netherlands) and maintained at the animal facility of the Zentrum für Zahn-, Mund- und Kiefergesundheit at the University of Würzburg under specific pathogen-free conditions. Mice were fed a standard rodent diet (Altromin Spezialfutter GmbH & Co. KG, Lage, Germany) and had free access to pathogen-free water. From the time when mice displayed paralytic signs, food and water were offered at ground level. All animal experiments complied with the German Law on the Protection of Animals and the “Principles of laboratory animal care” (NIH publication no. 86–23, revised 1985). The treatments were performed according to a protocol that was approved by the Regierung von Unterfranken, Germany (approval number 91/14).

### Induction and clinical assessment of EAE

The MBP-PLP fusion protein MP4 was obtained from Alexion Pharmaceuticals (Cheshire, CT, USA). By mixing mannide monooleate (Sigma-Aldrich, St. Louis, MO, USA) and paraffin oil (EMScience, Gibbstown, NJ, USA) at a ratio of 1:9, incomplete Freund’s adjuvant (IFA) was prepared. The addition of *Mycobacterium tuberculosis* H37 Ra (Difco Laboratories, Franklin Lakes, NJ, USA) at 5 mg/ml into IFA resulted in complete Freund’s adjuvant (CFA). Mice were immunized subcutaneously in both sides of the flank with 200 μg MP4 in 200 μl CFA. Mice received intraperitoneal injections of 200 ng pertussis toxin (List Biological Laboratories, Hornby, ONT, Canada) on the day of immunization and 48 h later. The clinical assessment was based on the standard EAE scoring system: (0) no disease, (1) floppy tail, (2) hind limb weakness, (3) full hind limb paralysis, (4) quadriplegia, and (5) death. Mice that were in between the clear-cut gradations of clinical signs were scored in increments of 0.5.

### Drug administration and grouping

MP4-immunized mice received a daily oral dose of 1 mg/kg body weight (BW) FTY720 (Sigma-Aldrich) diluted in 25% ethanol in *Aqua dest*. Control mice were MP4-immunized and treated with 20 μl of the vehicle solution only. Treatment was administered at the same time each day by pipetting the total volume of 20 μl into the mouth of each mouse as shown in Additional file 1. Treatment was started either at the peak of acute EAE or 50 days after EAE onset and was maintained for ~30 days except otherwise stated. For grouping of the mice, the scores at the beginning of the treatment were matched so that there was no significant difference between FTY720- and vehicle-treated mice (Additional file 2). Since the vehicle solution contained ethanol, we performed histological analysis on the liver to exclude any side effects due to the treatment (Additional file 1). We did not observe any signs of liver inflammation or fibrosis in MP4-immunized animals treated with FTY720 or vehicle solution, respectively.

### Tissue sampling

Blood from the tail vein was collected for flow cytometry. Mice were then sacrificed with CO_2_, blood from the inferior vena cava was used for ELISA, and draining inguinal lymph nodes from one side were dissected for ELISPOT analysis. The spleen was used for extended flow cytomery analysis. The animals were perfused with 4% paraformaldehyde (PFA, AppliChem, St. Louis, MO, USA). Afterwards, cerebella and inguinal lymph nodes from the other side were removed and embedded in paraffin.

### B cell enzyme-linked immunospot assay (ELISPOT)

MultiScreen®HTS 96-well ELISPOT plates (Merck Millipore, Darmstadt, Germany) were coated overnight with either 10 μg/ml MP4 (Alexion Pharmaceuticals, Inc.) or 15 μg/ml anti-mouse IgG (MabTech, Nacka Strand, Sweden), while coating with sterile PBS (Sigma-Aldrich) served as a negative control. Plates were blocked with 10% fetal bovine serum (FBS; Gibco, Thermo Fisher Scientific, Waltham, MA, USA) in sterile PBS at room temperature for 2 h. Inguinal lymph nodes were disintegrated mechanically and filtered through a 70 μm cell strainer (Corning Inc., Corning, NY, USA). Cells were washed twice with complete RPMI-1640 medium (Gibco), subsequently resuspended in HL-1 medium (Lonza, Basel, Switzerland) containing 1% L-glutamine (Sigma-Aldrich) and 1% penicillin/streptomycin (Sigma-Aldrich) and plated at 1 × 10^6^ cells/well followed by incubation at 37 °C and 7% CO_2_ for 24 h. Biotinylated goat anti-mouse IgG (Dako, Glostrop, Denmark) served as secondary antibody at 1:2000 dilution in 0.5% FBS/PBS + 0.025% Tween at 4 °C overnight. Plates were washed and incubated with streptavidin-alkaline phosphatase (Vector) at 1:800 dilution in 0.5% FBS/PBS for 2 h. Vector Blue substrate (Vector) was used for development. The spots were counted on an ImmunoSpot Series 6 UV Analyzer (CTL-Europe, Bonn, Germany).

### ELISA

ELISA plates (Nunclon Delta Surface; Nalge Nunc International, Rochester, NY, USA) were coated with MP4 at 3 μg/ml in PBS at 4 °C overnight. Plates were washed with PBS + 0.05% Tween and blocked with 1% milk powder (MP) in 0.05% PBS + 0.05% Tween at room temperature for 2 h. Serum samples were plated at 1:1000 dilution in 1% MP and incubated at 4 °C overnight. Negative controls contained 1% MP only. After renewed washing, biotinylated anti-mouse IgG (diluted 1:800 in 0.1% MP; eBioscience, San Diego, CA, USA) was added to the plates for overnight incubation at 4 °C. After incubation with avidin-horseradish peroxidase (diluted 1:1000 in 0.1% MP; BD Biosciences, San Jose, CA, USA) at room temperature for 2 h, plates were developed with tetramethylbenzidine (eBioscience). The reaction was stopped with 0.16 M sulfuric acid and read at 450 nm using a Perkin Elmer (Waltham, MA, USA) Victor 3 1420 Multilabel Counter with Wallac 1420 software version 3.00 revision 5.

### RNA-seq expression profiling

The complementary DNA (cDNA) libraries for all samples of isolated B cells were prepared using standard Illumina protocol, following the manufacturer’s instructions. B cells were purified from the spleens of MP4-immunized mice using a commercially available Mouse B Cell Isolation Kit (Miltenyi Biotec, Bergisch Gladbach, Germany). Subsequently, all libraries were sequenced with a high output 1 × 75 cycles kit using the Illumina Nextseq 500 sequencing platform that generated 75 bp long single-end reads. A total of ~400 million raw reads were produced that were processed and analyzed. The generated raw reads were processed using FastQC 0.11.3 for assessing read quality, amount of duplicates, and presence of adapter sequences. After this, the Illumina TruSeq adaptors were cleaved using cutadapt (version 1.12), and resulting reads were further trimmed keeping a quality drop value below a mean of Q20. Further, the processed sequences were mapped to the murine genome (mm12, GRCm38) using the short read aligner STAR-2.5.2b with genome and annotation files retrieved from GENCODE. For all the studied samples, the proportion of reads mapped to the mouse reference genome ranged between 86 and 89% in total. The sequences aligning to specific genes were quantified using bedtools subcommand intersect (version 2.15.0). Next, the differentially expressed genes were identified using DESeq2 (version 1.16.1). Only the genes having a Benjamini-Hochberg corrected *p* value below 0.05 were classified as significantly differentially expressed (DEGs). The data were visualized as MA plot using DESeq2’s function plotMA. To ascertain the biological relevance of the global transcriptomic differences between the sampling groups, KEGG-based enrichment analysis of DEGs was done using clusterProfiler. The RNA-seq data presented in this work has been deposited at the NCBI Gene Expression Omnibus and can be accessed through GEO series accession number GSE101753 (https://www.ncbi.nlm.nih.gov/geo/query/acc.cgi?acc=GSE101753).

### Flow cytometry of CD4^+^ T cells and CD19^+^ B cells in the blood

Blood of FTY720- and vehicle-treated mice was drawn from the tail vein and 40 μl of heparin were added. After erythrocyte lysis using an ammonium chloride-based red blood cell lysis buffer, cells were washed and incubated with BD Horizon™ Fixable Viability Stain 450 (FVS450; BD Biosciences, San Jose, CA, USA) at 4 °C for 30 min. Afterwards, cells were stained with anti-CD4 and anti-CD19 antibodies at 4 °C for 30 min (Additional file 3). Analysis was done on a FACS Canto™ II (BD Biosciences) at a flow rate of 2000 events per second, and each tube was run until 50,000 or 100,000 live events were recorded. Data were evaluated using FlowJo version 10.0.6 (Tree Star, Inc., Ashland, OR, USA). We excluded dead cells before a single gate on the FSC-H (forward scatter height)/FSC-A (forward scatter area) profile was set. Afterwards monocytes were excluded. B cells were characterized as CD19^+^CD4^−^ and T cells as CD19^-^CD4^+^ applying a CD4/CD19 bivariate gate. Gates were first set identically for all samples and adjusted individually according to unstained samples.

### Flow cytometry of S1P_1_^+^ T and B cells in lymph nodes and blood

Naïve female B6 mice were treated with 1 mg/kg BW FTY720 or vehicle solution for 10 consecutive days. Blood was obtained by cardiac puncture, and 5 μl of 0.5 M EDTA (AppliChem) was added. Erythrocyte lysis was performed using an ammonium chloride-based red blood cell lysis buffer. Lymph nodes were disintegrated mechanically and filtered through a 70 μm Falcon cell strainer (Corning Inc.). All samples were incubated with BD Horizon™ FVS450 (BD Biosciences) at 4 °C for 30 min. Afterwards, cells were stained with anti-CD4, anti-CD19, and anti-S1P_1_ antibodies at 4 °C for 30 min (Additional file 3). Analysis was done on a FACS Canto™ II (BD Biosciences) at a flow rate of 2000 events per second. Each sample was run until at least 10,000 (blood) or 100,000 (lymph nodes) live events were recorded. Data were evaluated using FlowJo version 10.0.6 (Tree Star, Inc.). We excluded dead cells before a single gate on the FSC-H (forward scatter height)/FSC-A (forward scatter area) profile was set followed by a single cell gate on the SSC-H (sideward scatter height)/SSC-A (sideward scatter area) profile. B cells were characterized as CD19^+^CD4^−^ and T cells as CD19^-^CD4^+^ applying a CD4/CD19 bivariate gate. Afterwards, S1P_1_^+^ T and B cells were identified. Gates were first set identically for all samples and adjusted individually according to unstained samples and fluorescence minus one controls (for S1P_1_).

### Flow cytometry of B cell subsets in the spleen

Spleens of FTY720- and vehicle-treated mice were disintegrated mechanically and filtered through a 70 μm Falcon cell strainer (Corning Inc.). After erythrocyte lysis using an ammonium chloride-based red blood cell lysis buffer, cells were washed and incubated with BD Horizon™ Fixable Viability Stain 780 (FVS780; BD Biosciences) at 4 °C for 30 min. Subsequently, samples were stained with the following anti-mouse antibodies at 4 °C for 30 min (Additional file 3): anti-CD5, anti-CD23, anti-CD43, anti-CD73, anti-CD80, anti-CD138, and anti-B220/CD45R. Analysis was performed on a FACS Canto™ II (BD Biosciences) at a flow rate of 2000 events per second, and each tube was run until 50,000 or 100,000 live events were recorded. Recorded data were evaluated using FlowJo version 10.0.6 (Tree Star, Inc.). We excluded dead cells before a single gate on the FSC-H (forward scatter height)/FSC-A (forward scatter area) profile was set. B220^+^ B cell subgroups were characterized as naïve B cells (CD43^−^CD73^−^CD80^−^CD138^−^), regulatory B cells (CD5^+^CD23^+/−^CD43^−^), B1a cells (CD5^+^CD23^−^CD43^+^), B1b cells (CD5^−^CD23^−^CD43^+^), and B memory cells (CD5^−^CD23^−^CD73^+^CD80^+^CD138^+/−^). To identify plasma cells (CD73^−^CD80^−^CD138^+^B220^−^), we set a B220/CD138 bivariate gate. Gates were first set identically for all samples and adjusted individually according to unstained samples and fluorescent minus one controls (for CD73, CD80, CD138).

### Flow cytometry of B cell subsets in the CNS

Periventricular cerebral tissue, cerebellum, and spinal cord of FTY720- and vehicle-treated mice were dissected and homogenized in 1 x HBSS^+/+^ (Gibco). After preparation of the stock isotonic Percoll, composed of Percoll^TM^ Plus (GE Healthcare) and 10 × HBSS^−**/**−^ (Gibco), the cell suspension was mixed with the stock to get a 30% solution. Additionally, a 70% solution was prepared by diluting the stock isotonic Percoll with 1 × HBSS^−/−^ (Gibco). To achieve a density gradient, the 30% solution was slowly pipetted onto the 70% solution and centrifuged at × 500*g* and 18 °C for 30 min without break. Cells were then isolated from the interlayer and washed in 1 × HBSS^+/+^ (Gibco). Afterwards, cells were resuspended in PBS and incubated with BD Horizon™ FVS780 (BD Biosciences) at 4 °C for 30 min. Subsequently, samples were stained with the following anti-mouse antibodies at 4 °C for 30 min (Additional file 3): anti-CD5, anti-CD23, anti-CD43, anti-CD73, anti-CD80, anti-CD138, and anti-B220/CD45R. Analysis was performed on a FACS Canto™ II (BD Biosciences) at a flow rate of 2000 events per second and each tube was run until 50,000 live events were recorded. Recorded data were evaluated using FlowJo version 10.0.6 (Tree Star, Inc.). We excluded dead cells before a single gate on the FSC-H (forward scatter height)/FSC-A (forward scatter area) profile was set. To identify plasma cells (CD138^+^ B220^−^) and B220^+^ B cells, we set a B220/CD138 bivariate gate. Gates were first set identically for all samples and adjusted individually according to unstained samples.

### Histological analysis (immunohistochemistry)

Lymph nodes were used as a control tissue and processed into 39 serial sections at 5 μm. The cerebella were cut into 84 serial sections at 5 μm and every eighth section was immunohistochemically stained with anti-B220/anti-CD3 antibodies in order to screen for the presence of inflammatory infiltrates. In case the sections were positive for B cell aggregates, the consecutive sections served for further characterization of lymphoid neogenesis using anti-CXCL13, -FDC-SP, -Ig, and -PNAd antibodies (Additional file 4). Hematoxylin/eosin stained sections were analyzed with ImageJ version 1.50b (National Institutes of Health, USA) to assess the size of the parenchymal area. The diaminobenzidine (DAB) staining protocol consisted of the following steps: paraffin-embedded sections were rehydrated and endogenous peroxidase was blocked. After citrate buffer-mediated epitope retrieval and protein blocking, the primary antibodies (Additional file 5) were incubated at 4 °C overnight. Biotinylated anti-rabbit, anti-rat, or anti-goat IgG (diluted 1:250 in PBS, Vector Laboratories, Burlingame, CA, USA) were used as secondary antibodies and incubated at room temperature for 1 h. For CD3/FDC-SP detection, the sections were additionally incubated with rabbit-peroxidase-anti-peroxidase complex (rb-PAP, Jackson ImmunoResearch Laboratories, West Grove, PA, USA, diluted 1:250 in PBS) for 30 min in the dark. The sections were developed with a horseradish peroxidase-based VECTASTAIN ABC Kit (Vector) and monitored under microscopic control in a solution containing nickel sulfate, glucose, ammonium chloride, DAB (Sigma-Aldrich), and glucose oxidase (Sigma-Aldrich). In case of CD3/B220 double staining, alkaline phosphatase-conjugated streptavidin (Vector) and Vector Blue Subtrate Kit (Vector) were additionally used. The sections were examined with a DM 2000 LED microscope (Leica, Wetzlar, Deutschland). B cell aggregates were classified by the aforementioned markers indicating lymphoid neogenesis. Positive staining of one marker categorized the aggregate as low-grade, of two or three markers as mid-grade, and the detection of all four markers as high-grade aggregate. Aggregates lacking all markers were identified as aggregates without lymphoid characteristics.

### Murine in vitro B cell aggregation assays

Spleens from 6 to 10-week-old naïve non-immunized female B6 mice (*n* = 6) were disintegrated mechanically and filtered through a 70 μm Falcon cell strainer (Corning Inc.) to obtain six individual samples. Cells were washed three times and resuspended in RPMI-1640 medium (Gibco). B cells were obtained through negative selection by using the Mouse B Lymphocyte Enrichment Set-DM (BD Biosciences) according to the vendor’s protocol and four wells with 5 × 10^5^ cells/well per mouse were plated onto a 96-well microplate (Greiner Bio-One, Frickenhausen, Germany). RPMI-1640 (Gibco) containing 1% L-glutamine (Sigma-Aldrich) and 1% penicillin/streptomycin (Sigma-Aldrich) was used as cell culture medium. FTY720 was added at a concentration of 8 μM. Vehicle solution at a corresponding volume was used as a control. After 90 min of pre-incubation at 37 °C and 7% CO_2_ on a shaker, cell activation was achieved by adding 0.25 μg/ml lipopolysaccharide (LPS, Sigma-Aldrich) and 5 U/ml recombinant murine interleukin-4 (IL-4, PeproTech, Rocky Hill, NJ, USA) followed by an incubation for 72 h. Well images were acquired with a Leica DM IL LED microscope (Leica) at ×200 magnification before FTY720 or vehicle solution was added, after 90 min of incubation and at the end of the 72 h incubation period. Images were evaluated using ImageJ Version 1.50b and its plugins ITCN (Centre for Bio-image Informatics, Santa Barbara, CA, USA) and Cell Counter (University of Sheffield, Sheffield, UK).

### Statistical analysis

GraphPadPrism 6.0 (GraphPad Software, Inc., La Jolla, CA, USA) was used for statistical analysis. Differences between groups were assessed using Mann-Whitney test. Three levels of statistical significance were differentiated: *p ≤ 0.05, **p ≤ 0.01 and ***p ≤ 0.001. Mean values and the standard error of the mean (SEM) are shown in the graphs.

## Results

### FTY720 treatment attenuates acute but not chronic EAE

**Fig. 1:**
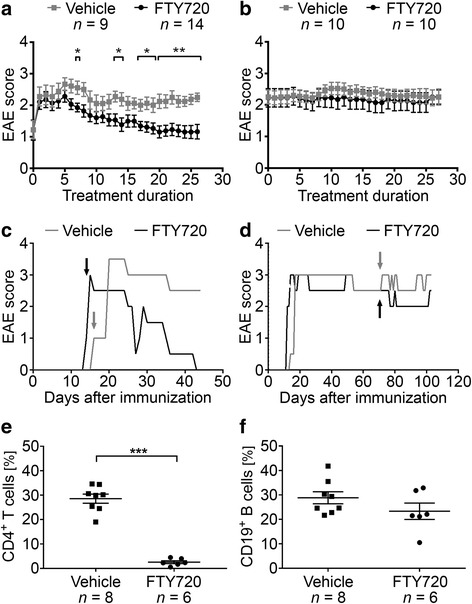
Disease course in mice treated with FTY720 either in the acute or chronic stage of EAE. MP4-immunized mice were treated with 1 mg/kg BW FTY720 or vehicle **a** at the peak of the disease or **b** 50 days after EAE onset. **c**, **d** Representative disease course of individual animals treated during **c** acute or **d** chronic EAE. *Arrows* indicate treatment onset. **e**, **f** Flow cytometry of CD4^+^ T cells and CD19^+^ B cells in the blood after treatment with FTY720 compared to vehicle. Mice were treated 50 days after disease onset. *p < 0.05, **p < 0.01, ***p < 0.001, Mann-Whitney test. *Bars* in **a** and **b** show mean values ± SEM

B6 mice were immunized with MP4 and received daily oral treatment with either 1 mg/kg BW FTY720 or vehicle, respectively, either at the peak of the disease or 50 days after disease onset. EAE onset in all treated cohorts was 19.05 ± 3.20 days post immunization, and treatment started at a mean EAE score of 2.19 ± 0.19 in the FTY720 group compared to a mean EAE score of 2.29 ± 0.19 in the vehicle group (*p* = 0.90). Detailed information on the disease course are provided in Additional file 2. FTY720 treatment at the peak of disease significantly attenuated clinical EAE as early as day 7 of application (Fig. 1a, c). This significant amelioration of the disease course was continuously seen from day 17 on until the end of the experiment, which was 27.65 days (±1.09 days) after treatment onset. In contrast, FTY720 treatment 50 days after onset of disease did not result in any significant differences in EAE scores when compared to the control group (Fig. 1b, d). To determine whether treatment with FTY720 triggered the drug-related reduction in peripheral lymphocyte counts, we performed flow cytometry of CD4^+^ T cells and CD19^+^ T cells on tail vein blood that was collected at end of the treatment period. The proportion of CD4^+^ T cells was significantly reduced in the peripheral blood of FTY720-treated mice (*n*_FTY720_ = 6, *n*_vehicle_ = 8 with *p* < 0.001), while the percentage of CD19^+^ B cells was not significantly altered (*p* = 0.18, Fig. 1e, f). One explanation for the diminished effect of FTY720 on B cells could be lower S1P_1_ receptor expression compared to T cells. However, our results rather indicate that there were no differences in the extent of S1P_1_ receptor expression on T and B cells in blood and lymph nodes of FTY720- and vehicle-treated mice (Additional file 6).

### FTY720 treatment neither affects the MP4-specific B cell and antibody response nor B cell function in general

**Fig. 2:**
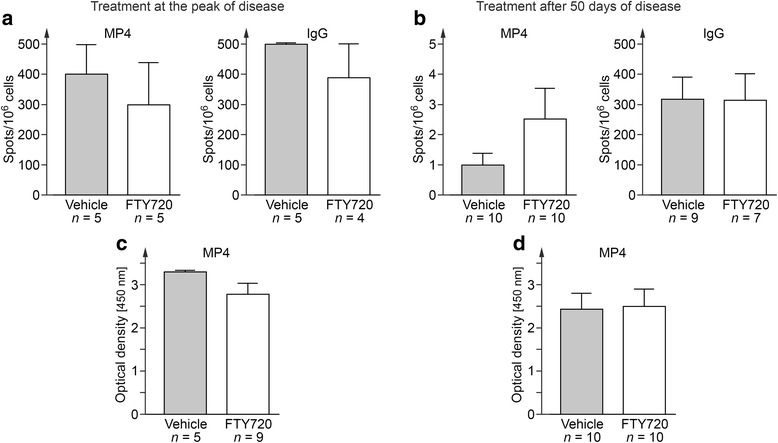
Measurements of MP4-specific B cells and antibody production. B cell ELISPOT was performed on draining inguinal lymph nodes of MP4-immunized mice to detect total or MP4-specific IgG during **a** acute or **b** chronic EAE. MP4-specific serum antibodies were measured by ELISA during **c** acute or **d** chronic treatment. Mice were treated either with 1 mg/kg BW FTY720 or vehicle. *Bars* show means ± SEM

Next, we set out to study whether there was a potential impact of FTY720 on B cell function. To this end, we collected serum and draining inguinal lymph nodes either at the end of the acute or the chronic treatment period and performed measurements of MP4-specific antibody production by ELISPOT and ELISA. Fig. 2 shows that there was no difference in the number of total or MP4-specific IgG producing B cells in FTY720- and vehicle-treated mice (Fig. 2a, b). Comparable results were obtained for the detection of MP4-specific antibodies (Fig. 2c, d).

**Fig. 3:**
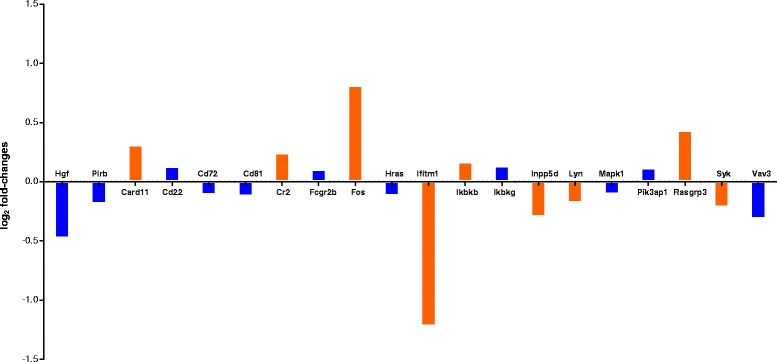
RNA sequencing analysis of isolated B cells from the spleens of MP4-immunized B6 mice that were treated with FTY720 (*n* = 5) or vehicle (*n* = 4), respectively, at the peak of EAE. Differential expression analysis results for B cell-related genes are shown. Log_2_ fold-changes of the comparison between vehicle- vs*.* drug-treated samples (*n* = 3 replicates each) calculated by DESeq2 for genes associated with BCR signaling in KEGG are plotted. Results with an adjusted *p* value below 0.05 are colored in *orange* while the remaining ones are colored in *blue*

In a next step, we investigated the effect of FTY720 on B cell gene expression profiles by performing RNA sequencing (Fig. 3). To this end, we purified B cells from the spleens of MP4-immunized mice that were treated at the peak of EAE with either FTY720 (*n* = 5) or vehicle (*n* = 4). Analysis was performed for genes that are relevant for B cell receptor (BCR)-mediated signaling. When focusing on genes that showed a log_2_ fold-change and had a *p* value below 0.05 some differences between FTY720- and vehicle-treated mice were evident. On the one hand, there was a significant downregulation of Lyn and Syk, which are involved in the transmission of signals from the BCR [21]. In addition, Ifitm1 expression was decreased after FTY720 treatment. Ifitm1 has been described to reduce the threshold of BCR engagement needed for cell stimulation in conjunction with interactions involving CD81 and the BCR [22]. On the other hand, we observed an upregulation of factors involved in apoptotic cell death including Card11 [23], Fos [24] and Rasgrp3 [25] suggesting pro-apoptotic effects of FTY720.

### Effects of FTY720 on B cell emigration from SLOs

**Fig. 4:**
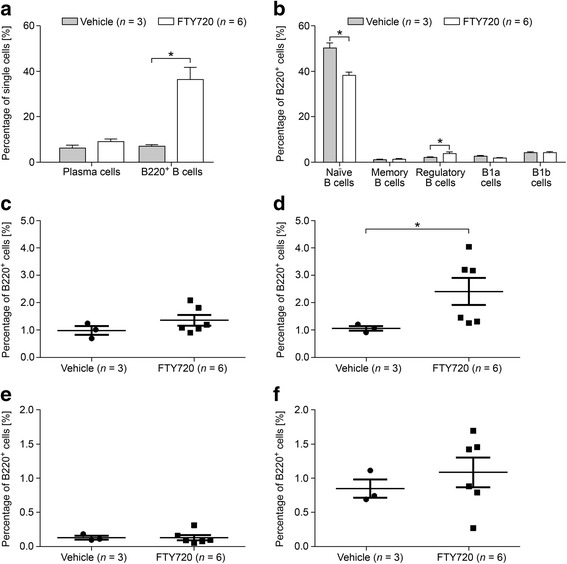
B cell subsets were measured by flow cytometry in the spleens of MP4-immunized mice that were treated at the peak of disease with 1 mg/kg BW FTY720 or vehicle, respectively. **a** Percentages of plasma cells and of B220^+^ B cells. **b** Percentages of the different B220^+^ B cell subsets. More detailed analysis of the percentages of **c** CD23^+^ regulatory B cells, **d** CD23^−^ regulatory B cells, **e** CD138^+^ memory B cells, and **f** CD138^−^ memory B cells. *Bars* show means ± SEM. *p < 0.05, Mann-Whitney test

**Fig. 5:**
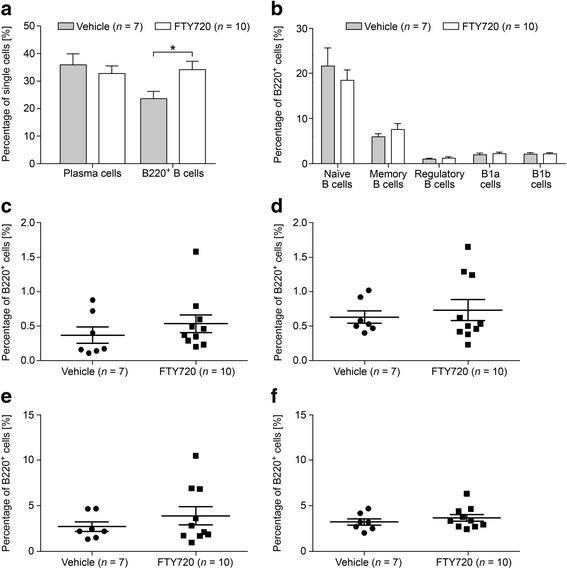
B cell subsets were measured by flow cytometry in the spleens of MP4-immunized mice that were treated 50 days after EAE onset with 1 mg/kg BW FTY720 or vehicle, respectively. **a** Percentages of plasma cells and B220^+^ B cells. **b** Percentages of the different B220^+^ B cell subsets. More detailed analysis of the percentages of **c** CD23^+^ regulatory B cells, **d** CD23^−^ regulatory B cells, **e** CD138^+^ memory B cells, and **f** CD138^−^ memory B cells. *Bars* show means ± SEM. *p < 0.05, Mann-Whitney test

To study the effect of FTY720 on B cells in greater detail, we performed flow cytometry on spleen cells of MP4-immunized mice that were treated either with FTY720 (*n* = 6) or vehicle (*n* = 4) for 22.90 ± 1.83 days at the peak of disease (Fig. 4) or for 30.71 ± 0.16 days during chronic EAE (50 days after EAE onset) (*n*_FTY720_ = 10; *n*_vehicle_ = 7) (Fig. 5). Mice that received treatment at the peak of disease showed significantly altered percentages of total B220^+^ B cells and of naïve B cells. Vehicle-treated animals harbored 7.09% B220^+^ B cells compared to 36.42% in FTY720-treated animals (*p* = 0.02). While a mean percentage of 49.70% naïve B cells was observed in vehicle-treated animals, FTY720-treated mice showed a significant decrease to 38.60% (*p* = 0.02). Furthermore, we detected a significant increase in the percentage of CD23^−^ regulatory B cells after treatment with FTY720 (compare 1.07 to 2.31%; *p* = 0.02) (Fig. 4). In mice that were treated after 50 days of EAE, a significant increase was observed only for the overall population of B220^+^ cells. While vehicle-treated mice possessed 23.63% B220^+^ cells, this percentage increased to 34.13% in the FTY720 group (*p* = 0.03) (Fig. 5). To investigate the effects of FTY720 on B cell infiltration into the CNS of MP4-immunized mice, flow cytometry was performed on the brain, cerebellum, and spinal cord of mice that were treated at the peak of EAE with either vehicle (*n* = 4) or FTY720 (*n* = 6). Due to the low number of cells, detailed subset analysis could not be performed, but there were no differences in the percentages of plasma cells and of B220^+^ B cells comparing the two groups (Additional file 7).

### Treatment with FTY720 affects the development but not maintenance of B cell aggregates in the CNS

**Fig. 6:**
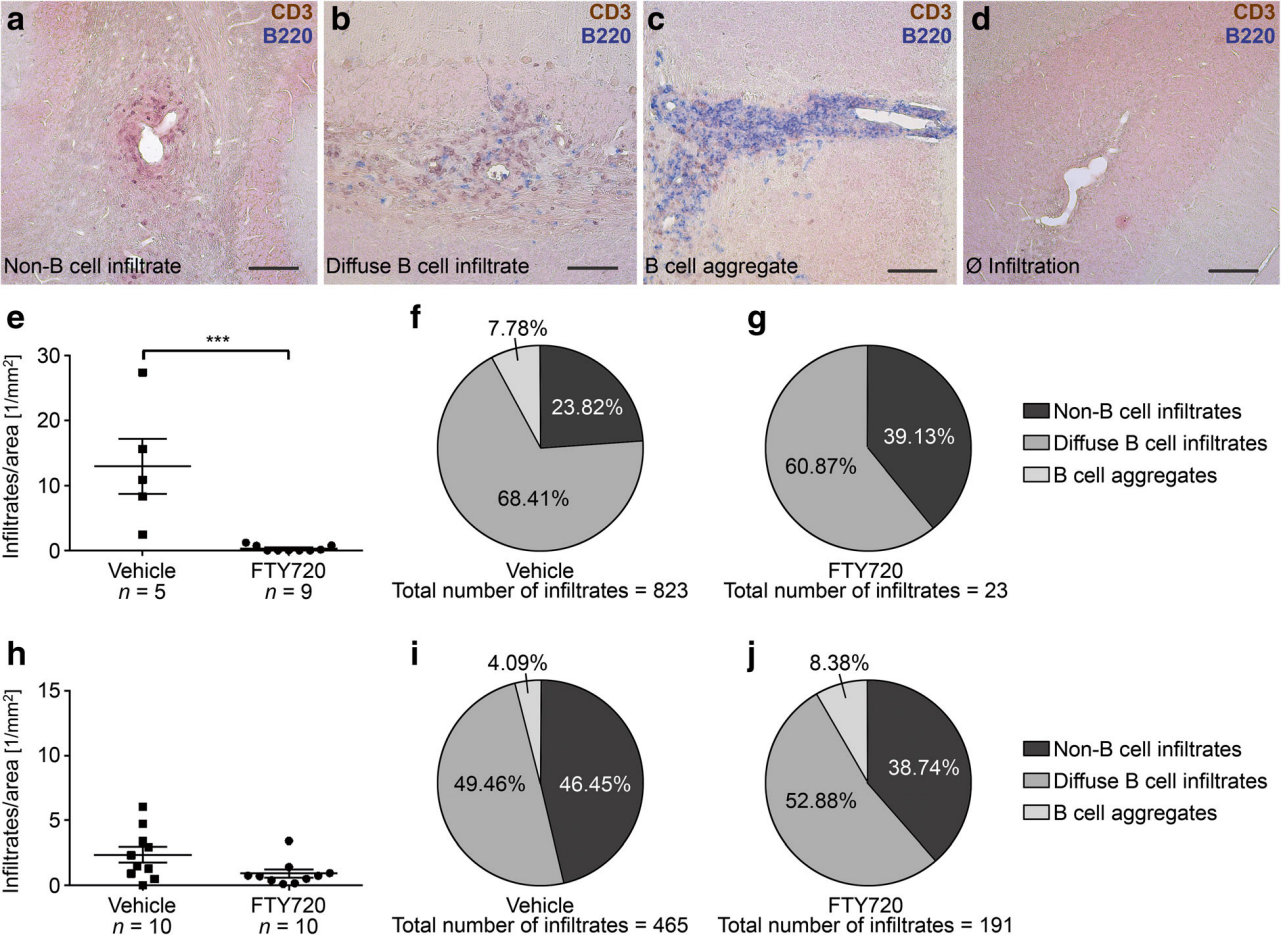
Histological analysis of B cell aggregates in the cerebella of MP4-immunized mice. **a–d** CD3/B220 double staining of representative cerebellar sections of a mouse treated with vehicle solution at the peak of disease. The panels demonstrate the different infiltrate categories. *Bars* represent 100 μm. **e**–**g** Quantification of the number of infiltrates per parenchymal area and distribution of infiltrate categories in mice treated either with 1 mg/kg BW FTY720 or vehicle at the peak of disease. **h**–**j** Quantification of the number of infiltrates per parenchymal area and distribution of infiltrate categories in mice treated 50 days after disease onset. Graphs show means ± SEM. *p < 0.05, **p < 0.01, Mann-Whitney test

In addition to investigating the effects of FTY720 treatment on the peripheral B cell response, we also explored the impact of FTY720 on B cell aggregation in the CNS. To this end, the cerebella of MP4-immunized mice were assessed after ~30 days of treatment, which started either at the peak of acute EAE or 50 days after disease onset. Overall, three different kinds of inflammatory infiltrates were investigated (Fig. 6a–d): non-B cell infiltrates, diffuse B cell infiltrates, or B cell aggregates. Non-B cell infiltrates were characterized by perivascular CD3^+^ T cells in a loose arrangement, while similar structures with presence of additional B220^+^ B cells were defined as diffuse B cell infiltrates. B cell aggregates showed a tight and compartmentalized perivascular cluster of cells that contained at least one third B cells. Mice treated with FTY720 during acute EAE showed a significant reduction in the overall number of inflammatory infiltrates per parenchymal area (Fig. 6e; *p* = 0.001). This significant decrease also pertained to the different categories of inflammation (non-B cell infiltrates: *p* = 0.006; diffuse B cell infiltrates: *p* = 0.001; B cell aggregates: *p* < 0.001). Interestingly, in five of the nine mice treated during acute EAE we did not find any infiltrates, and in none of the remaining four mice B cell aggregates were detected. In contrast, mice which were treated after 50 days of EAE did not show a significant difference in the total number of infiltrates per parenchymal area (*p* = 0.075), diffuse B cell infiltrates (*p* = 0.123) or B cell aggregates (*p* = 0.978), when compared to the vehicle-treated group. However, there was a significant reduction in the number of non-B cell infiltrates per area in FTY720-treated mice (*p* = 0.007). B cell aggregates were further subdivided into B cell aggregates with or without evidence for lymphoid neogenesis based on the expression of CXCL13 and the presence of follicular dendritic cells (FDC), plasma cells (Ig^+^), and/or PNAd^+^ high endothelial venules (HEV). Since no B cell aggregates were detected in mice treated with FTY720 during acute EAE, the analysis of lymphoid neogenesis was limited to the vehicle group. All four characteristics found in lymphoid tissue-like structures were observed except for PNAd^+^ HEVs. As a consequence, mid-grade aggregates were the most common infiltrates at this stage of EAE. Overall, >70% of B cell aggregates showed features of lymphoid neogenesis. In mice treated after 50 days of disease with vehicle solution, all B cell aggregates displayed markers of lymphoid follicle-like structures and one high-grade B cell aggregate was observed. Interestingly, after treatment with FTY720 in chronic EAE, the absence of high-grade aggregates, a higher number of low-grade aggregates and the presence of an aggregate without any lymphoid features indicated an attenuating effect of FTY720 on lymphoid neogenesis. Statistical significance was reached for the reduction in mid-grade aggregates in FTY720- compared to vehicle-treated animals (*p* = 0.04). The differences in TLO marker distribution are summarized in Additional file 8.

### No direct effect of FTY720 on B cell aggregation in vitro

**Fig. 7:**
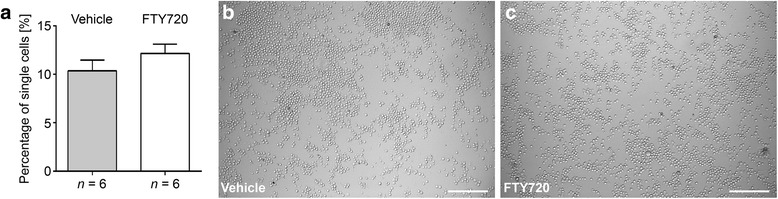
In vitro B cell aggregation assay. B cells were purified from naïve B6 mice and pre-incubated with 8 μM FTY720 or corresponding vehicle solution for 90 min. B cells were then stimulated with 0.25 μg/ml LPS and 5 U/ml IL-4 for 72 h to induce aggregation. **a** Percentage of single cells at the end of the culture period. Graphs show means ± SEM. **b**, **c** Representative images of B cell aggregation under each of the two culture conditions. *Scale bars* represent 100 μm

Using an in vitro assay, we further investigated a potential direct effect of FTY720 on B cell aggregation*.* B cells were purified from *n* = 6 naïve non-immunized B6 mice, pre-incubated with 8 μM FTY720 or vehicle for 90 min, and aggregation was subsequently induced using a combination of LPS and IL-4. The percentage of single cells was determined after 72 h of incubation. As shown in Fig. 7, there was no difference comparing FTY720- and vehicle-treated B cells after in vitro aggregation.

## Discussion

The aim of our study was to investigate the therapeutic effect of FTY720 on B cells in the B cell-/antibody-dependent MP4-EAE model. We employed two different treatment strategies: mice were either treated at the peak of EAE or during chronic disease. Our readout parameters of interest comprised global effects of FTY720 on B cells in addition to effects on MP4-specific antibody production, B cell subset distribution in SLOs, and B cell aggregation in the CNS.

FTY720 is an orally administered S1P receptor modulator (functional antagonist) and currently approved for the treatment of RR-MS. Its main mode of action is thought to be mediated by internalization and degradation of the S1P_1_ receptor on lymphocytes, consequently trapping them in SLOs [26, 27]. As a consequence, less autoreactive lymphocytes are able to migrate into the CNS and to participate in inflammatory and neurodegenerative processes [28, 29]. While effects of FTY720 on T cells have been described in detail [30–32], only limited data are available on the effects on B cells. In-depth studies on B cell-specific effects of FTY720, however, are of outmost importance considering the crucial role of B cells in the pathogenesis of MS and the recent most promising advances in the development of B cell-depleting therapies [3–5, 33]. In particular, the notion that the development of B cell aggregates and the consecutive evolvement into TLOs is a hallmark feature of SP-MS and at least partly linked to clinical disease progression [14] demands further studies on the effect of FTY720 on these structures.

In a first set of experiments, we explored the effects of FTY720 on peripheral B cell function. Measuring both MP4-reactive and total IgG production, we did not detect any differences between FTY720- or vehicle-treated mice. These data are in line with early experiments examining the immunosuppressive effect of FTY720 in the context of organ transplantation [20, 34]. Pinschewer et al*.* investigated the humoral response of B6 mice in a viral infection model, showing that FTY720 did not affect B cell antibody secretion [34]. Furthermore, they measured the vesicular stomatitis virus antibody titer after immunization and transient FTY720 treatment and did not find a difference compared to the untreated control animals. This led to the conclusion that B memory cells were not impaired by the drug [34]. Activation, proliferation, and effector functions of human B cells were also not influenced by FTY720 treatment [20]. However, it should be noted that Grützke et al*.* proposed a direct effect of FTY720 on B cell migration, as they found an increased migratory activity in in vitro BBB experiments comparing B cells from FTY720-treated MS patients with those of untreated controls [35].

In our study, RNA sequencing revealed differential effects of FTY720 on isolated B cells from MP4-immunized mice. These included the downregulation of Lyn, Syk, and Ifitm1 indicating the modulation of BCR-mediated signaling [21] as well as a potential pro-apoptotic mode of action of the drug [23–25]. Pro-apoptotic effects of FTY720 on B cells have been described before–however, it was also noted that this effect was much less pronounced in B cells compared to T cells, most likely due to the fact that B cells have more abundant anti-apoptotic protein Bcl-2 [36].

When performing B cell subset analysis by flow cytometry, we found that the percentage of CD4^+^ T cells was significantly diminished in the peripheral blood, while CD19^+^ B cell numbers were not. On the one hand, these data are in line with the aforementioned relative resistance of B cells to apoptosis induction by FTY720 [36] and also with a study published by Matloubian et al*., * who proposed that CD19^+^ B cells are less dependent on S1P_1_-mediated egress than CD4^+^ T cells [27]. On the other hand, our data partly contradict the significant decrease of CD19^+^ B cells in human PBMCs of MS patients receiving FTY720 [37–39] –partly because we still detected a significantly increased number of total B220^+^ B cells in the spleen, which suggests a B cell-specific effect of FTY720 although this effect might not have been visible to its full extent in the peripheral blood. Interestingly, in previous reports, FTY720 treatment had only little impact on the number of B cells in the CSF, the extent of intrathecal IgG synthesis, and the presence of OCBs [38]. Also, in our study, there was no difference in the number of B cells and plasma cells in the CNS comparing vehicle- and FTY720-treated mice. When analyzing different B cell subsets, FTY720-treated MS patients showed an increase in CD19^+^IgD^+^CD27^−^ naïve B cells in the peripheral blood [35]. In line with these data, we noticed that B220^+^CD43^−^CD73^−^CD80^−^CD138^−^ naïve B cells were significantly decreased in the spleen after FTY720 treatment in MP4-induced EAE. Furthermore, Grützke et al. detected a strongly increased ratio of regulatory B cells in the peripheral blood of MS patients [35]. In our model, the percentages of splenic CD23^−^ regulatory B cells were increased in mice treated with FTY720 at the peak of disease, but not in chronic EAE. Since these cells might represent marginal zone-bound regulatory B cells that are permanently located in the white pulp of the spleen, this increase could be explained by activation caused by the inflammatory milieu [40]. Other B cell subsets remained unaffected in FTY720-treated animals. Since there is no sufficient data on S1P receptor expression on murine lymphocyte subsets, we have no final explanation for potential discrepancies between our data and the results obtained in the human studies. However, it should be noted that we used a more detailed panel of extracellular markers for flow cytometry experiments than in the human studies. To our knowledge, we are the first to offer extensive data on the influence of FTY720 on splenic B cell subsets in a B cell-dependent EAE model. To investigate S1P_1_ receptor expression on T and B cells after treatment with FTY720, we conducted flow cytometry analyses of lymph nodes and blood and detected no significant differences. Yet, S1P_1_^+^CD4^+^ T cells and in particular S1P_1_^+^CD19^+^ B cells were elevated in the blood compared to the lymph nodes, which is in line with the results of Matloubian et al. [27]. In addition, Sinha et al. [41] suggested that S1P-mediated chemotaxis is of minor importance for B cell egress from SLOs. Their proposed model of B cell lymph node transit describes a rather complex interaction of S1P_1_ and S1P_3_ and the overall activation status of the cell, which might also explain the lack of effect of FTY720 treatment on peripheral B cell numbers.

The most remarkable effect of FTY720 in our study was achieved when treatment was initiated during acute EAE. Treatment during acute EAE both significantly ameliorated clinical EAE and prevented the formation of B cell aggregates in the CNS. To determine whether the effect on B cell aggregates was due to a direct effect on B cells or rather a “bystander” phenomenon, we performed in vitro assays on purified mouse B cells demonstrating a lack of inhibitory effect of FTY720 on B cell aggregation. Since not only B cell aggregates but also global cerebellar inflammation was significantly reduced after FTY720 treatment, we suggest that the inhibition of T cell infiltration was a key reason for the absence of B cell aggregate formation in the CNS in the acute treatment cohort. Peters and colleagues have previously shown that T_H_17 cells are required for B cell aggregate formation [42], and we have demonstrated the presence of T_H_17 cells in the vicinity of TLOs in the MP4 model [11]. Along these lines, FTY720 was shown to significantly suppress the ability of T cells to be polarized to T_H_17 in vitro [43]. Interestingly, there was no effect of FTY720 on the maintenance of already developed B cell aggregates in chronic EAE. However, we noted a significant reduction in the evolution of B cell aggregates into TLOs, suggesting that the drug may either directly affect lymphoid neogenesis through yet unknown pathways or that T_H_17 cells may also be required for TLO evolution.

It is currently debated whether CNS B cell aggregates need constant lymphocyte supply from the periphery. Studies investigating the effects of the anti-VLA4 antibody natalizumab have reported that OCBs indicating local antibody production vanished in a proportion of natalizumab-treated patients [44]. If one assumes that OCBs are at least in part associated with TLOs as local source for plasmablasts, it is conceivable that blockage of the BBB by natalizumab [45, 46] triggered TLO disruption. Our data, however, rather suggest that TLOs are inert and stable structures that are not directly dependent on supply from the outside once established. Along these lines, there are currently no substantial data on any of the approved MS therapies and on autologous hematopoetic stem cell transplantation that they have an effect on OCBs in the CSF. If TLOs are indeed stable, it will be highly relevant to determine whether TLOs are actually required or important for disease perpetuation.

## Conclusions

Taken together, this study used MP4-induced EAE as a B cell- and antibody-dependent model of MS to explore potential B cell-specific effects of FTY720. The data suggest that these effects pertained to the modulation of BCR signaling, a decreased egress of B220^+^ B cells from SLOs and to a decrease in the evolution of CNS B cell aggregates into TLOs. Since the next generation drug siponimod is currently being tested in SP-MS with promising results, future studies will have to elucidate whether this drug has even more pronounced effects on B cells and B cell aggregates and whether such effects are beneficial for both the treatment of RR- and SP-MS.

## Additional files

Additional file 1: Oral treatment of mice with vehicle or 1 mg/kg BW FTY720 (a). (b) Macroscopic image of the liver after 30 days of treatment with vehicle solution. (c) HE staining of the liver and (d) higher magnification of the image. (e) Macroscopic image of the liver after 30 days of treatment with FTY720. (f) HE staining of the liver and (g) higher magnification of the image. Scale bars represent 200 μm in (c) and (f) and 100 μm in (d) and (g). Results are representative of *n* = 10 mice treated with vehicle solution and *n* = 12 mice treated with FTY720. (TIF 10745 kb)

Additional file 2: Clinical parameters of EAE in mice treated either with FTY720 or vehicle. (DOCX 16 kb)

Additional file 3: Antibodies used for flow cytometry. (DOCX 16 kb)

Additional file 4: B cell aggregates in the cerebellar parenchyma show different characteristics of lymphoid neogenesis. (a) HE staining, (b) CD3/B220 double staining, (c-f) CXCL13, FDC-SP, Ig, and PNAd DAB staining. Scale bars represent 100 μm. (TIF 11019 kb)

Additional file 5: Antibodies used for immunohistochemistry. (DOCX 59 kb)

Additional file 6: Flow cytometry of S1P_1_^+^ T and B cells. Cells were measured in the blood (a) and lymph nodes (b) of naïve B6 mice that were treated either with 1 mg/kg BW FTY720 or vehicle for 10 consecutive days. Bars show means ± SEM. (TIF 102 kb)

Additional file 7: Flow cytometry analysis of plasma cells and of B220^+^ B cells in the CNS. Cells were measured in periventricular brain, cerebellar, and spinal cord tissue of MP4-immunized mice that were treated at the peak of disease with either 1 mg/kg BW FTY720 or vehicle. Bars show means ± SEM. (TIF 62 kb)

Additional file 8: TLO marker distribution in mice treated with vehicle at the peak of disease (a, b). Treatment with vehicle (c, d) or FTY720 (e, f) was initiated 50 days after EAE onset. (TIF 387 kb)

## Abbreviations

B6: C57BL/6
BBB: Blood-brain-barrier
BCR: B cell receptor
BW: Body weight
CNS: Central nervous system
CSF: Cerebrospinal fluid
CXCL13: Chemokine ligand 13
DAB: Diaminobenzidine
DEG: Differentially expressed gene
EAE: Experimental autoimmune encephalomyelitis
FDC: Follicular dendritic cell
HE: Hematoxylin and eosin
HEV: High endothelial venule
Ig: Immunglobulin
LPS: Lipopolysaccharide
IL-4: Interleukin-4
MBP: Myelin basic protein
MOG: Myelin oligodendrocyte glycoprotein
MP: Milk powder
MP4: MBP-PLP fusion protein
MS: Multiple sclerosis
OCB: Oligoclonal bands
PBS: Phosphate-buffered saline
PFA: Paraformaldehyde
PLP: Proteolipid protein
PNAd: Peripheral node addressin
RR-MS: Relapsing-remitting MS
SLO: Secondary lymphoid organ
SP-MS: Secondary progressive MS
TLO: Tertiary lymphoid organ
