# Supplementary figures and images for: Differential effects of FTY720 on the B cell compartment in a mouse model of multiple sclerosis
Source: J Neuroinflammation. 2017 Jul 24;14:148. doi: 10.1186/s12974-017-0924-4 (PMC5525315; doi:10.1186/s12974-017-0924-4)

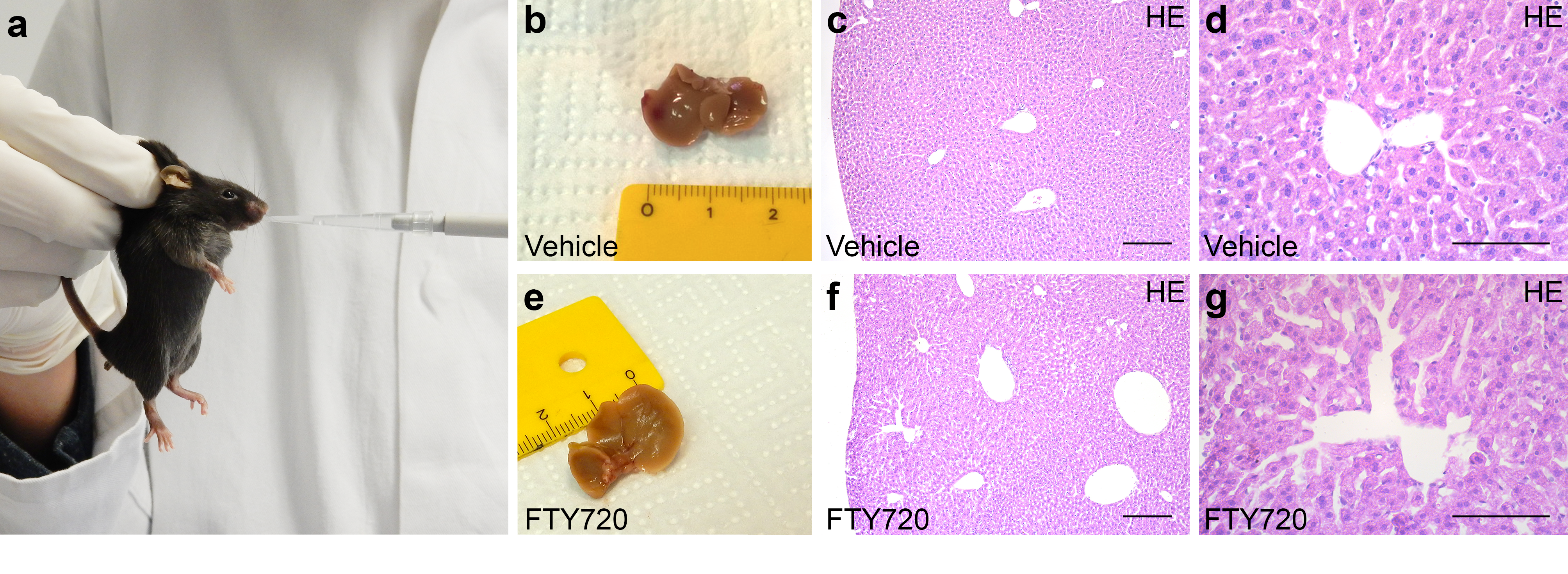

Supplement: Supplementary file 1 — Oral treatment of mice with vehicle or 1 mg/kg BW FTY720 (a). (b) Macroscopic image of the liver after 30 days of treatment with vehicle solution. (c) HE staining of the liver and (d) higher magnification of the image. (e) Macroscopic image of the liver after 30 days of treatment with FTY720. (f) HE staining of the liver and (g) higher magnification of the image. Scale bars represent 200 μm in (c) and (f) and 100 μm in (d) and (g). Results are representative of n = 10 mice treated with vehicle solution and n = 12 mice treated with FTY720. (TIF 10745 kb) [file 12974_2017_924_MOESM1_ESM.tif]

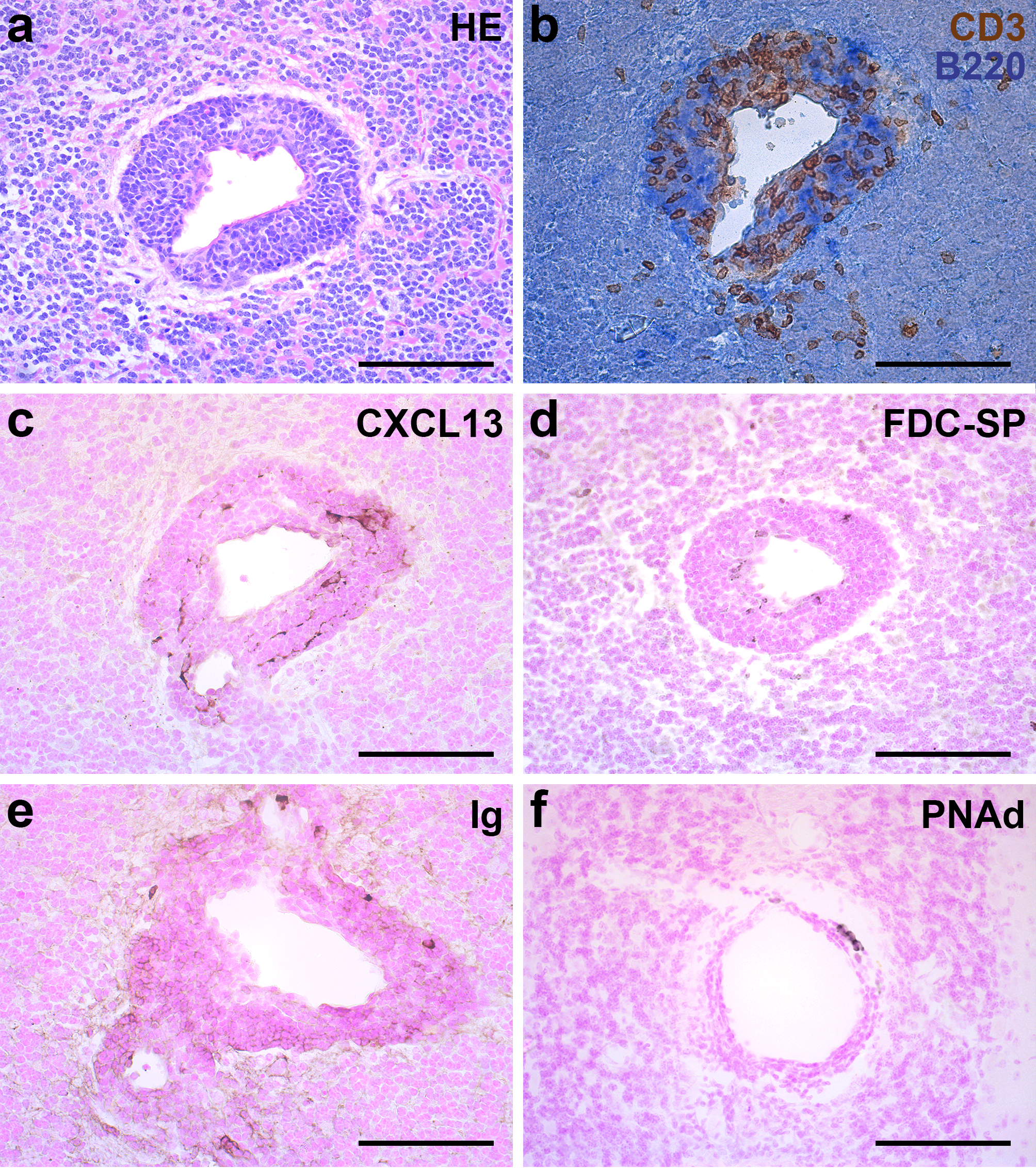

Supplement: Supplementary file 4 — B cell aggregates in the cerebellar parenchyma show different characteristics of lymphoid neogenesis. (a) HE staining, (b) CD3/B220 double staining, (c-f) CXCL13, FDC-SP, Ig, and PNAd DAB staining. Scale bars represent 100 μm. (TIF 11019 kb) [file 12974_2017_924_MOESM4_ESM.tif]

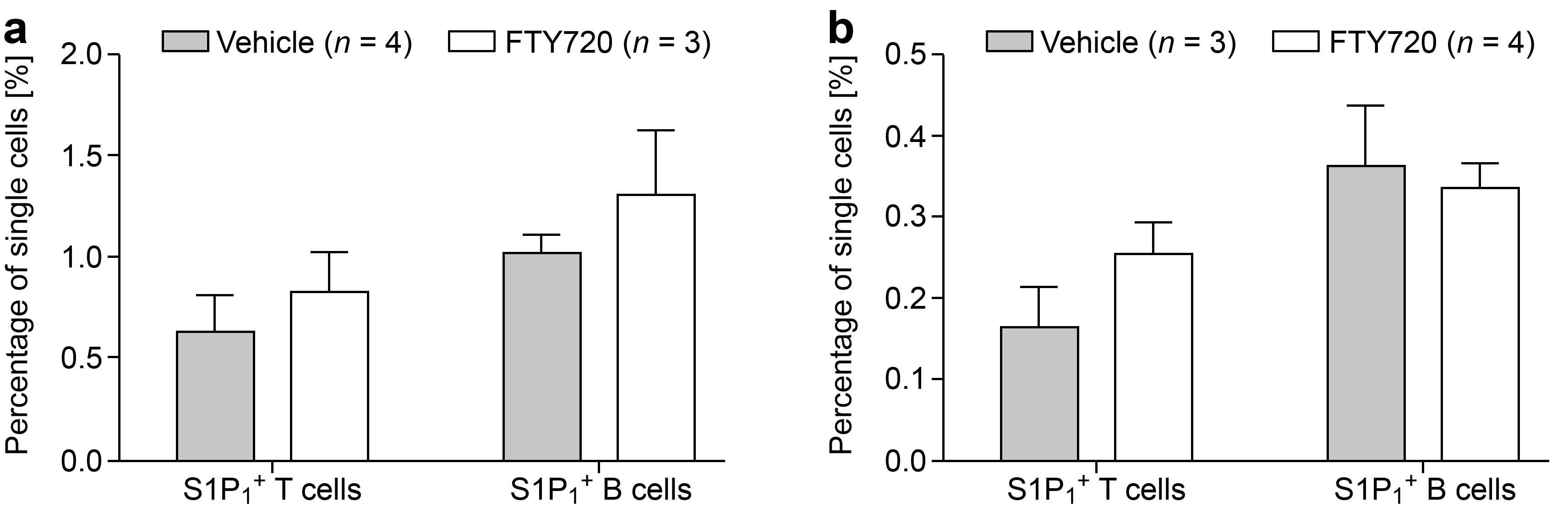

Supplement: Supplementary file 6 — Flow cytometry of S1P1 + T and B cells. Cells were measured in the blood (a) and lymph nodes (b) of naïve B6 mice that were treated either with 1 mg/kg BW FTY720 or vehicle for 10 consecutive days. Bars show means ± SEM. (TIF 102 kb) [file 12974_2017_924_MOESM6_ESM.tif]

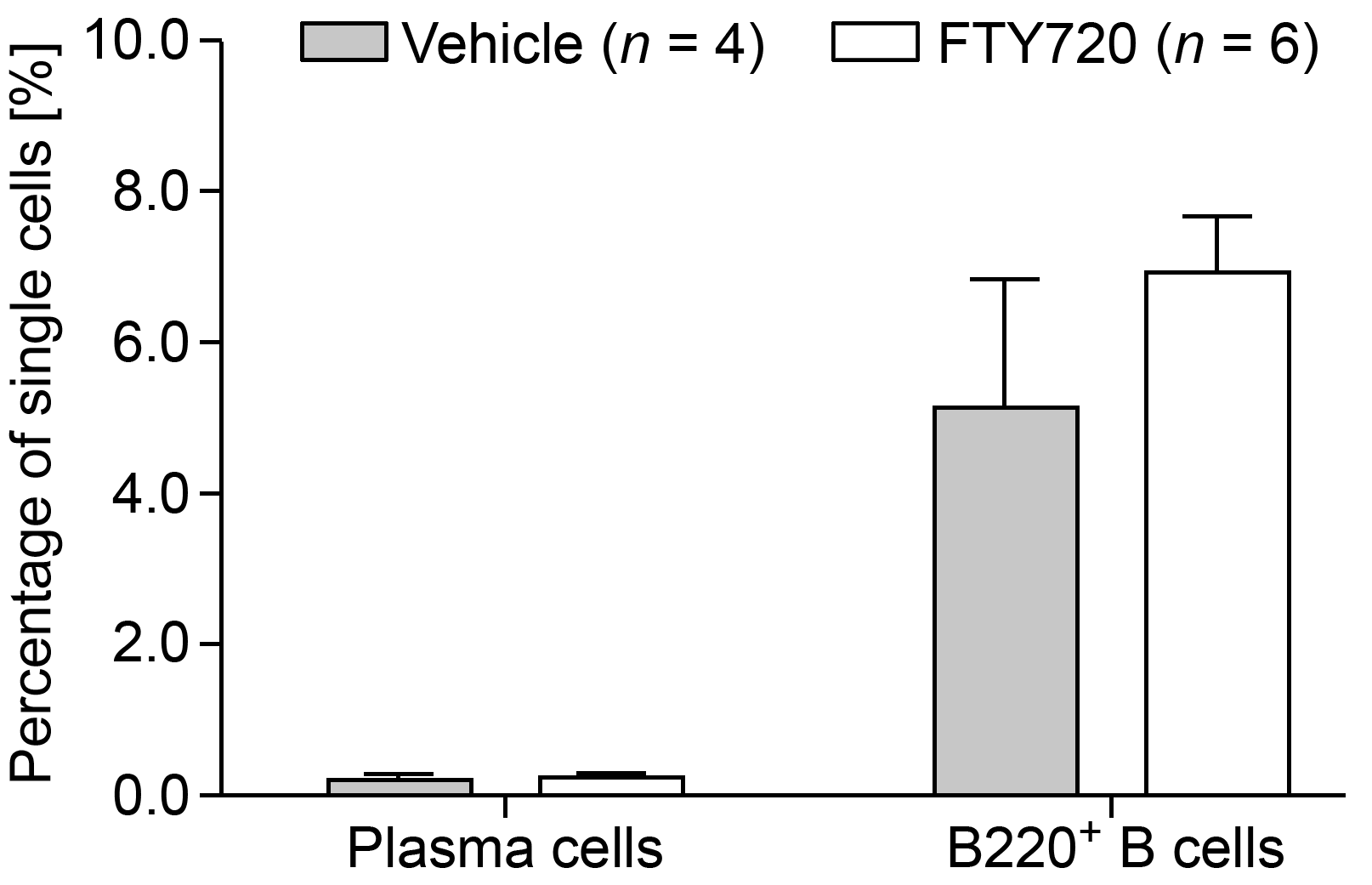

Supplement: Supplementary file 7 — Flow cytometry analysis of plasma cells and of B220+ B cells in the CNS. Cells were measured in periventricular brain, cerebellar, and spinal cord tissue of MP4-immunized mice that were treated at the peak of disease with either 1 mg/kg BW FTY720 or vehicle. Bars show means ± SEM. (TIF 62 kb) [file 12974_2017_924_MOESM7_ESM.tif]

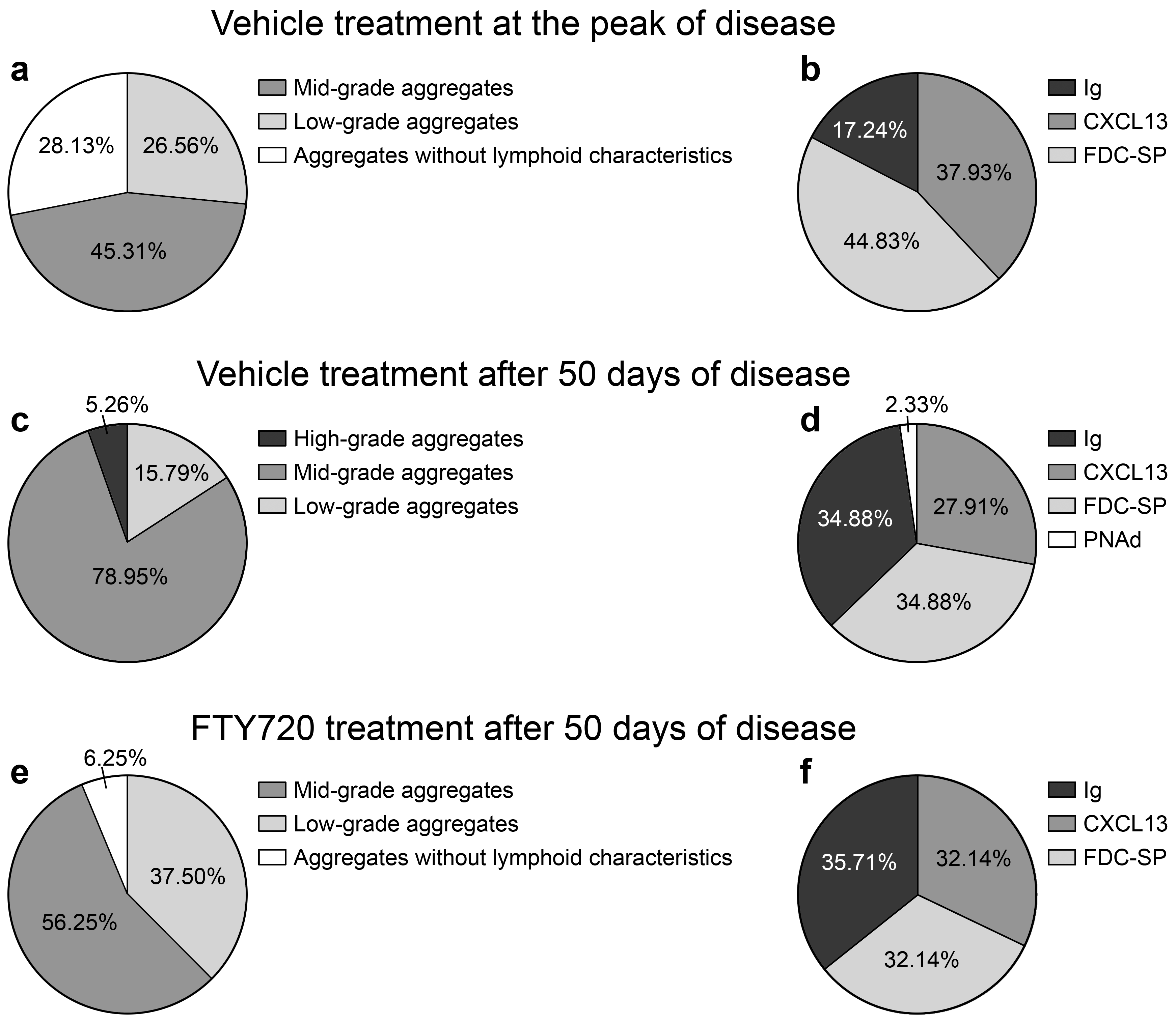

Supplement: Supplementary file 8 — TLO marker distribution in mice treated with vehicle at the peak of disease (a, b). Treatment with vehicle (c, d) or FTY720 (e, f) was initiated 50 days after EAE onset. (TIF 387 kb) [file 12974_2017_924_MOESM8_ESM.tif]
