# Supplementary material for: Differential effects of FTY720 on the B cell compartment in a mouse model of multiple sclerosis
Source: J Neuroinflammation. 2017 Jul 24;14:148. doi: 10.1186/s12974-017-0924-4 (PMC5525315; doi:10.1186/s12974-017-0924-4)
Supplement: Supplementary file 2 — Clinical parameters of EAE in mice treated either with FTY720 or vehicle. (DOCX 16 kb) [file 12974_2017_924_MOESM2_ESM.docx]

**Additional file 2:** Clinical parameters of EAE in mice treated either with FTY720 or vehicle.

**Treatment at the peak of EAE**

**EAE onset Score at Treatment Final score Score difference**

(days after **EAE onset duration**

immunization)

**Vehicle**

*n* = 9 16.44 ± 2.06 2.23 ± 0.30 27.00 ± 1.90 2.22 ± 0.15 0.06 ± 0.35

**FTY720**

*n* = 14 16.43 ± 0.74 2.11 ± 0.24 28.07 ± 1.34 0.82 ± 0.22 1.29 ± 0.17

***p*-value** 0.28 0.82 0.81 < 0.01 < 0.01

All data are shown as mean values ± SEM.

**Treatment after 50 days of EAE**

**EAE onset Score at Treatment Final score Score difference**

(days after **EAE onset duration**

immunization)

**Vehicle**

*n* = 10 15.30 ± 2.79 2.30 ± 0.75 29.0 ± 1.83 2.20 ± 0.89 0.10 ± 0.15

**FTY720**

*n* =10 15.20 ± 4.84 2.30 ± 0.98 29.0 ± 1.83 2.20 ± 0.89 0.10 ± 0.18

***p*-value** 0.35 0.83 > 0.99 0.92 0.90

All data are shown as mean values ± SEM.
