# Supplementary material for: Differential effects of FTY720 on the B cell compartment in a mouse model of multiple sclerosis
Source: J Neuroinflammation. 2017 Jul 24;14:148. doi: 10.1186/s12974-017-0924-4 (PMC5525315; doi:10.1186/s12974-017-0924-4)
Supplement: Supplementary file 3 — Antibodies used for flow cytometry. (DOCX 16 kb) [file 12974_2017_924_MOESM3_ESM.docx]

**Additional file 3:** Antibodies used for flow cytometry.

**Antibody Host species Fluorochrome Clone Origin**

Anti-mouse CD4, Rat PerCP-Cy™ 5.5 RM4-5 BD Biosciences,

monoclonal San Jose, CA, U.S.A.

Anti-mouse CD5, Rat PerCP 53-7.3 BD Biosciences,

monoclonal San Jose, CA, U.S.A.

Anti-mouse CD19 Rat APC 6D5 BioLegend,

San Diego, CA, U.S.A.

Anti-mouse CD23, Rat Alexa Fluor® 647 B3B4 BD Biosciences,

monoclonal San Jose, CA, U.S.A.

Anti-mouse CD43, Rat PE-Cy™7 S7 BD Biosciences,

monoclonal San Jose, CA, U.S.A.

Anti-mouse CD45R/ Rat BV510 RA3-6B2 BD Biosciences,

B220, monoclonal San Jose, CA, U.S.A.

Anti-mouse CD73, Rat PE TY/23 BD Biosciences,

monoclonal San Jose, CA, U.S.A.

Anti-mouse CD80, Hamster BV421 16-10A1 BD Biosciences,

monoclonal San Jose, CA, U.S.A.

Anti-mouse CD138, Rat BB515 281-2 BD Biosciences,

monoclonal San Jose, CA, U.S.A.

Anti-mouse S1P_1_/ Rat PE 713412 R&D Systems,

EDG-1, monoclonal Minneapolis, MN, U.S.A.
