# Supplementary material for: Differential effects of FTY720 on the B cell compartment in a mouse model of multiple sclerosis
Source: J Neuroinflammation. 2017 Jul 24;14:148. doi: 10.1186/s12974-017-0924-4 (PMC5525315; doi:10.1186/s12974-017-0924-4)
Supplement: Supplementary file 5 — Antibodies used for immunohistochemistry. (DOCX 59 kb) [file 12974_2017_924_MOESM5_ESM.docx]

**Additional file 5**: Antibodies used for immunohistochemistry.

**Primary antibodies Host species Dilution Clone Origin**

Anti-mouse CD3, Rabbit 1:100 SP7 Abcam, Cambridge, MA, U.S.A.

monoclonal

Anti-mouse CD45R/ Rat 1:500 RA3-6B2 eBioscience, San Diego, CA

B220, monoclonal U.S.A.

Anti-mouse CXCL13/ Goat 1:80 N/A R&D Systems, Minneapolis,

BLC/BCA-1, MN, U.S.A.

polyclonal

Anti-mouse FDC-SP, Rabbit 1:500 N/A Bioss, Woburn, MA, U.S.A. polyclonal

Anti-mouse Ig, Goat 1:1000 N/A Dako, Glostrup, Denmark

biotinylated,

polyclonal

Anti-mouse PNAd Rat 1:200 MECA-79 BD Biosciences, San Jose, CA, monoclonal U.S.A.

**Secondary antibodies**

Anti-rabbit IgG, Goat 1:250 N/A Vector Laboratories,

biotinylated Burlingame, CA, U.S.A.

Anti-Rat IgG Goat 1:250 N/A Vector Laboratories,

biotinylated Burlingame, CA, U.S.A.

Anti-Goat IgG Rabbit 1:250 N/A Vector Laboratories,

biotinylated Burlingame, CA, U.S.A.
